# Ambivalent Agents: The Social Mobility Industry and Civil Society Under Neoliberalism in England

**DOI:** 10.1111/1468-4446.70026

**Published:** 2025-09-08

**Authors:** Anna Mountford‐Zimdars, Louise Ashley, Eve Worth, Christopher James Playford

**Affiliations:** ^1^ School of Education University of Exeter Exeter UK; ^2^ School of Business and Management QMUL London UK; ^3^ Archaeology and History University of Exeter Exeter UK; ^4^ Social and Political Sciences, Philosophy, and Anthropology, University of Exeter Exeter UK

**Keywords:** civil society, higher education, inequality, marketisation, neoliberalism, polanyi, social mobility

## Abstract

This article examines civil society organisations working to enhance social mobility in England, especially through higher education. Against the backdrop of neoliberal governance, we investigate whether these organisations operate as protective counter‐movements resisting marketisation or as institutional mechanisms that stabilise the inequalities they aim to address. Drawing on Karl Polanyi's concept of the ‘double movement’ and Nancy Fraser's critique of marketised social protections, we map and analyse over 100 charities and non‐profits established since 1992. We combined qualitative coding of organisational websites across nine Fraserian dimensions with Latent Profile Analysis to identify structural patterns within the field. Findings reveal that most organisations balance critical framings of inequality with funder‐compatible, technocratic delivery models. We argue this structural ambivalence is a defining feature of civil society under neoliberalism and show how the social mobility industry operates to suggest symbolic reform without redistributive transformation. Our contribution is threefold: we provide the first systematic typology of the UK's social mobility sector, extend Polanyi and Fraser's theoretical frameworks into social mobility and education policy, and offer a methodological model combining qualitative and quantitative methods with AI‐assisted research.

## 1 Introduction

Neoliberalism is a contested and increasingly polarising term, marked by ongoing debate about its meaning and analytical utility (Davies 2014). Here, we use the term to refer to a governing rationality that shifts the role of the state from the direct provision of social goods such as education, housing, and welfare, towards market management and the delegation of responsibility to private actors and civil society organisations. Since its consolidation in UK policy over 4 decades ago, a substantial body of scholarship has traced this transformation in state functions (Newman and Clarke 1997). Alongside this shift, researchers have noted a parallel development: namely, that as the state retreats from directly addressing social problems, a range of civil society institutions, including charities, NGOs, and private firms, have stepped in to fill the gap (Clarke 2004; Eikenberry and Kluver 2004; Jessop 2007).

These developments have been analysed using Karl Polanyi's (1944) concept of the ‘double movement,’ in which the expansion of market logics prompts protective responses from society, often mediated through civil institutions. Published in 1944, Polanyi's work has since been used by scholars to help theorise the contradictions of neoliberal capitalism and the failure of market self‐regulation, albeit with modifications from his original stance. Especially relevant here, Polanyi argued the state could act as a correction and protection against market forces, while more recent scholarship has suggested the state is often complicit as protective responses have themselves been marketized (Fraser 2011, 2017), and civil society organisations are increasingly co‐opted into neoliberal logics (F. Block and Somers 2014; Peck 2010; F. L. Block 1990; Brie 2019).

Our specific aim is to consider the nature and characteristics of the UK's ‘social mobility industry’ for what this reveals about the transformation of protection under neoliberal governance and the implications for Polanyian theory. To provide brief context, for over 2 decades social mobility has enjoyed cross‐party consensus as a preferred strategy for tackling social inequality, particularly focussing on long‐range upward mobility, moving people from the ‘bottom’ of society to the ‘top’ as judged by highly remunerated jobs (Payne 2017). While a matter of concern to politicians across the industrialised West, the focus on social mobility has been especially intense in the UK, arguably reflecting several factors peculiar to the national context, including a political desire to offer symbolic solutions to inequality without addressing its structural cause (Ingram and Gamsu 2022). This focus has been accompanied by the development of a network of private and third sector actors seeking to promote mobility through a range of interventions, though often centring on higher education (Payne 2017, Todd 2021). While some individuals have been supported in making upward mobility journeys as a result, this has had a limited impact at the macro level, and according to some studies, overall rates of upward mobility are at their lowest for 50 years (IFS 2023).

In critical literature, the cause of such failures has been extensively analysed, and often blamed on a political emphasis on individual ‘merit,’ which helps reinforce class‐based exclusion while having a limited impact on wider structural inequalities (Ashley 2022; Breen and In 2023; Ingram and Gamsu 2022; Sandel 2020). Polanyian concepts have been applied less often to specific policy arenas yet we contend that this ‘social mobility industry’ (Payne 2017, Todd 2021) represents an ideal opportunity to trace how protective mechanisms are actively constructed, constrained, and potentially co‐opted in practice, helping in turn to generate a more granular, empirical understanding of how market logics are negotiated, institutionalised, and legitimated within particular fields.

To do so, first we mapped almost 150 civil society actors established since 1992 and working in social mobility in England.^1^ Drawing particularly on Fraser's (2011, 2017) critique of marketised social protections and focussing specifically on charities and non‐profits as emblematic of civil society, and an important domain from which counter‐movements have traditionally been expected to emerge, we then combined qualitative coding of organisational websites across nine dimensions with Latent Profile Analysis (LPA), to identify structural patterns within the field. Our analysis highlights important differences between organisations in this space, to suggest civil society is not universally co‐opted, as some room exists for structurally more critical organisations. However, resistance is partial or hybrid, and it is more common overall for organisations in this space to actively participate in stabilising the neoliberal order, albeit inadvertently.

Our study offers an original theoretical contribution by bringing together political economy, theories of civil society, and critical policy analysis to examine the social mobility agenda as an example of neoliberal legitimation. Our study confirms Fraser's (2011, 2017) argument that even progressive civil society may be co‐opted to stabilise market systems but also adds clarity by demonstrating the *mechanisms* of co‐option, which we suggest take place through factors such as funding dependencies, metric‐led delivery and professionalised governance. These insights into how market‐conforming and market‐resisting logics are negotiated, not just in policy but also in organisational practice and form, are important for both theorists of neoliberalism and practitioners seeking to understand the political limits of progressive reform given considerable structural constraints. Before expanding on our findings, we review literature on neoliberalism, civil society, and social mobility, with particular attention to debates about the co‐optation of resistance and the role of third sector organisations in policy delivery.

### 1.1 Neoliberalism and Civil Society

The rise and persistence of neoliberalism has often been understood through the lens of Karl Polanyi's (1944) analysis of market society. Though responsible for a large body of work, Polanyi (1944) set out his core arguments in his book, ‘*The Great Transformation,*’ which describes how in modern capitalism, the economy becomes ‘disembedded’ from society and social relations, shifting the role of the markets from serving social needs, to society serving markets. Critically, this free‐market society is the product of a political project, requiring state intervention. In the process, land, labour and money become what Polanyi called ‘fictitious commodities,’ treated as products for sale like all other commodities, suggesting a dereliction of human nature and needs. His notion of the ‘double movement’ describes how, as markets cause social harms, such as poverty, insecurity and inequality, society fights back, through demands for protection, regulation and welfare. For Polanyi, this form of (re)‐regulation and decommodification was expected to be largely managed by the State, yet civil society also plays a crucial role as the posited location for starting counter movements, envisaged as taking place via workers' organisations, charities, social reformers and alternative organisational forms, such as co‐operatives.

Given its influence, it is not surprising that Polanyi's work has also been subject to critique. One argument is that the ‘double movement’ is too binary, where this suggests a pendulum‐like dynamic between market expansion and social protection, therefore oversimplifying the complex, overlapping relationships between markets and social institutions (Beckert 2009). Burawoy (2010) argues for example that Polanyi placed too much hope in the countermovement as a protectionist force with one implication that, following the first ‘great transformation’ during the nineteenth century's industrial revolution, he failed to predict the market fundamentalism of the late twentieth century.

More critical interpretations challenge the assumption of neutrality in both state and civil society, arguing that counter‐movements themselves can be shaped by, and entangled within, the ideological logics of neoliberalism (Goodwin 2021). The work of Nancy Fraser (2014, 2017) has been especially influential here, as she points out that neoliberalism involves a strong, proactive state that constructs and enforces markets, often in authoritarian ways. Her critique develops a quasi‐Polanyian version of his ideas (Fraser 2011, 140), where she also argues that the binary suggested by Polanyi between ‘disembedding’ and protective counter movements is too simple, but introduces a third dynamic: emancipatory movements, defined as efforts to overcome domination and hierarchy, such as struggles for gender equality and racial justice. These are characterised as not simply *reactions* to markets, to secure protection, but also attempts to *change* society and challenge oppression. A related insight is that emancipatory demands such as autonomy and recognition have been co‐opted into neoliberal agendas, or ‘hijacked’, and turned into justifications for market friendly reforms. One example is privatising welfare in the name of empowerment. This leads to what Fraser (2017) calls ‘marketized social protections:’ forms of social provision claiming to protect against inequality or exclusion but doing so via market logics.

Fraser's (2011, 2017) arguments have something in common with earlier contributions in the same broad tradition. For example, F. L. Block (1990) earlier explored how the state manages discontent by fostering civil society organisation within strict neoliberal movements, selectively supporting certain kinds of civil society which help markets function better. Two decades later, Peck (2010) underlined that neoliberalism does not mean no state but instead an *active* state that reorganises society around markets, ideas encapsulated within the notion of ‘roll‐out neoliberalism,’ where civil society organisations, non‐profits, and private actors are mobilised to manage social fallout from market driven policies, such as mass inequality. Collectively, these contributions extend Polanyi's notion of the ‘market’ as a political project, needing state regulation and direction (Burawoy 2010, 302), where the state is not seen as a balancing force between market and society but as an active site of class and ideological struggle. They also offer important insights into how and why policies and initiatives have apparently contradictory goals, to reinforce neoliberal norms even as they claim social justice. It is against this background that we consider how social mobility policy and practice may function as part of this contradiction.

While not always using this terminology, the post‐war period in the UK has seen two waves of especially intense academic and political interest in social mobility: the first in the 1960s and 1970s when an expansion of professional and managerial jobs contributed to a growth in absolute social mobility and associated changes to patterns of social stratification (Goldthorpe 2013, 2016). The second is often identified as originating from the mid‐2000s to the present day, a period which includes the revival of a broader research agenda exploring patterns and experiences of social mobility (Dodd et al. 2017). Policy interest was evidenced a little earlier by the release of a series of White Papers and reports. Towards the start of this agenda, notable examples were the Labour government's ‘*New Opportunities’* report in 2009. The Liberal Democrat's Independent Commission on Social Mobility published *Through the Glass Ceiling* in 2008, while the coalition government published *Opening Doors, Breaking Barriers* in 2011.

A common theme running through this agenda is the positioning of higher education as a primary location where upward mobility is expected to be realised. This has been pushed as a key idea by successive administrations since the 1990s, starting with Tony Blair's ‘new’ Labour administration target of 50% of young people in higher education in 1999, by ensuring participation became available to a wider demographic. Related reforms to schools and tertiary education were explicitly justified to promote social mobility, aims taken‐up by later Conservative administrations (Payne 2017). Especially relevant to our analysis is the incremental privatisation of formerly state‐owned services and the introduction of market forces to tackle societal challenges, operationalised in higher education via the introduction of quasi‐markets (Le Grand and Bartlett 1993). In theory, market mechanisms prioritised within this system are expected to lead to efficiency, while also contributing to more equitable outcomes. In practice, this has not been the effect. As one example, increases in higher education participation among under‐represented groups is disproportionately channelled into lower tariff institutions with less positive graduate outcomes, a phenomenon described elsewhere as ‘effectively maintained inequality’ (Lucas 2001).

The mass expansion of higher education and its democratisation has not then made a significant contribution to higher rates of social mobility in the UK, despite an entire industry aiming to support related goals (Payne 2017). While these failures are now widely recognised, our specific aim here is to consider the nature and characteristics of this industry, and its mechanisms, offering an illusion of change while reinforcing the legitimacy of neoliberalism. Before expanding on these findings, we describe our research methods next.

### 1.2 Research Methods

As researchers with significant experience in relevant fields, we were aware of the existence of a ‘social mobility industry’ as we started our research. However, our first task was to map the organisations comprising this industry systematically. As such, between September 2022 and April 2024 our team searched for publicly available websites to find all organisations currently in operation tasked as some or all of their mission to support social mobility, with a particular focus on higher education. Our analysis extended to organisations founded since 1992, the year of the Higher Education Act. This was an important moment, when arguably the terminology of social mobility became more common in policy and media discourses (Mandler 2016).

We used the following process for identifying organisations. First, members of the research team with direct experience in higher education social mobility initiatives each listed organisations based on their personal experiences and knowledge. Second, this team undertook internet searches using variations of the key words ‘social mobility’; ‘higher education’, ‘Charity’ and ‘higher education access.’ Stages one and two yielded 123 organisations. Third, we held a brainstorming session with a further 40 practitioners which yielded an additional 13 organisations and, using artificial intelligence (ChatGPT), found an additional 6 organisations. Finally, four human sector experts were enlisted to check our master list and again, found 6 organisations.

We included organisations with a national reach and restricted the analysis to England, as education is a matter for the four devolved administrations in the UK. We excluded the 296 individual higher education providers as, while mandated to deliver social mobility, they typically do so by working with external organisations. In total, this data‐mapping exercise led us to identify 147 organisations operating in the social mobility space. We classified these organisations according to their main remit, dividing them between seven types: campaign groups representing 9.5% of the total (e.g. Fair Education Alliance); charities representing 52% of the total (e.g. Sutton Trust); higher education provider networks representing 9% (e.g. Russell Group); non‐profit organisations representing 8% (e.g. The Bridge Group); registered companies representing 7% (e.g. wonkHE); UK government bodies representing 17% (e.g. Office for Students) and other HE social mobility networks, that provide services to HE providers at 15% (e.g. Higher Education Access Tracker).

### 1.3 Data Analysis

We were initially struck by the sheer number of organisations in this space, which showed a particular uptick over the past decade, so that there is now one social mobility organisation for every eight of the 3444 secondary schools in England. A more recent expansion of this sector is likely to reflect key events such as the publication of the Cabinet Office Panel for Fair Access to the Professions in 2009, which reported on class‐based exclusions from professions such as law and accountancy, following which The Social Mobility Commission was established in 2012, to act as the government's critical friend on related matters, and provide research and thought leadership.

However, beyond the sheer size of this industry, we also wanted further insights into its nature and characteristics, leading to our specific research question, derived from Polanyian theory: *To what extent do these organisations operate as counter‐movements resisting commodification or as institutional mechanisms that help to stabilise the very inequalities they are meant to address*?

To address this question, we narrowed our focus to charities and non‐profits as key components of civil society, which are notionally also tasked by the State with buffering individuals and communities from the effects of commodification in education and labour. We approached data analysis aware that the answer to our research question was likely to vary across a range of dimensions. To identify what dimensions would best capture this complexity, building from our literature review, we initially devised four categories: ‘delegated responsibility from state to civil society’; ‘marketisation and professionalisation of social justice’; ‘depoliticization’; and ‘legitimising neo‐liberalism through progressive performance and language.’ We randomly selected a sample group of organisations (*n* = 5) and coded their language and activities under all four dimensions, with 1 being limited conformity and 3 being high. We used organisational websites as our main data source because they are accessible public facing records, offering good insights into features such as vision and aims, history, target users, funders and impact and outcome measures.

This first round of coding was completed by the first three authors, working independently. Having conferred on results, the team agreed a wider range of dimensions was needed, since the original four did not capture the full variation in how organisations positioned themselves in relation to areas such as market logic or structural critique.

**FIGURE 1:**
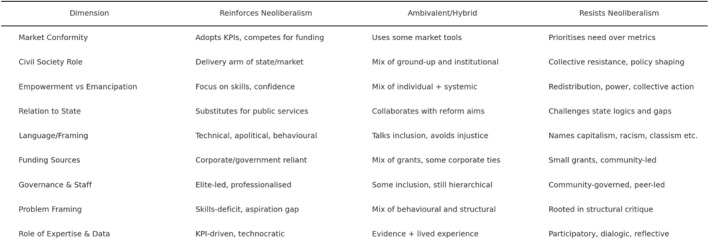
Dimensions and framework for coding social mobility organisations.

Since we were working with a large dataset, to help us identify and differentiate between categories, and conduct subsequent coding, we used ChatGPT, a large language model (LLM) using artificial intelligence (AI). ChatGPT was first released in 2022. As such, and as is true for AI more generally, this is a relatively novel tool to conduct qualitative analysis, though one that is becoming increasingly popular. Previous researchers have pointed to related risks and limitations. One is that AI makes mistakes and may struggle to produce findings which allow for contextual depth and consistency (Yue et al. 2025). These risks underline that coding and categorisation must take place with close human assistance but, with these caveats, studies have found ChatGPT can generate coding results that enhance efficiency and scalability, yet are similar to manual approaches (Pattyn 2025; Prescott et al. 2024). With these points in mind, we prompted ChatGPT to suggest additional dimensions for coding. What followed was an iterative process, moving between AI generated suggestions, and discussions between the authors about their suitability and value. We also used suggested dimensions to manually code data from our original sample group. After several rounds, we settled on a total of nine dimensions, which particularly reflect Fraser's (2011) critique of the co‐option of civil society under neoliberalism, and which we felt allow for a nuanced assessment of how organisations simultaneously navigate funding pressures, governance structures, and ideological commitments. These dimensions are listed in Figure 1, with brief definitions.

The next stage was to test the strength of these dimensions and associated definitions as we instructed ChatGPT to perform the coding exercise on a sample group of 10 organisations, using a three‐point scale: Red (3) suggesting on that dimension the organisation reproduces neoliberal logic; Amber (2) suggesting it was ambivalent/mixed; Green (1) the organisation resists neoliberalism. The lead authors conducted the same exercise using the same sample group. Overall, human and AI coding produced similar results, though ChatGPT was slightly more generous in its interpretation. As one example, ChatGPT was more likely to code organisations as ‘amber’ under ‘Empowerment versus. Emancipation,’ compared to the lead authors, who were slightly more likely to code the same organisations as ‘red.’ Where we found differences such as these, we challenged ChatGPT to explain the logic. On this occasion, it responded that an organisation is coded red under this heading only if a focus on empowerment reinforces or masks neoliberal logics, to individualise structural problems, depoliticise inequality and divert energy away from collective action or critique. Amber would imply on the other hand that the organisation focuses a little more on personal development, access or motivation, without explicitly challenging structures, organising collectively, or redistributing power. We were comfortable with this interpretation, recognising that as human coders we were being a little less optimistic (and also that different human coders could produce different interpretations, depending for example on whether they are rooted in more critical or policy‐centric models). The interpretation by ChatGPT was also highly consistent, a significant advantage when coding this large dataset.

Once this validation process was complete, we tasked ChatGPT to perform the coding exercise for the entire dataset, while continuing to check results as they were produced. This process allowed us to construct a spreadsheet, with the results for each organisation colour coded across each dimension (spreadsheet available as a data supplement on request from corresponding author). Consistent with initial results, this heatmap was predominantly amber, with some red, and less green, tending to suggest a hybrid between reproduction and resistance overall, though with the emphasis on the former.

**FIGURE 2:**
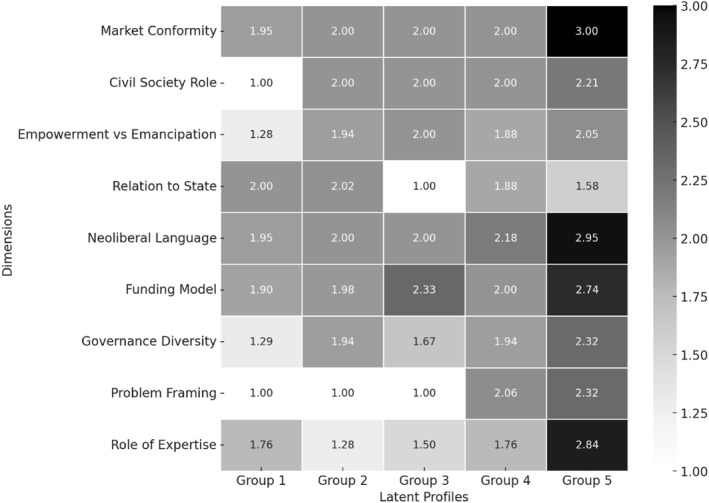
Latent profiles for 5 group model (marginal means), *n* = 110.

Since we consider ambiguity a structural feature of the field, this represents an important finding, as we will explain. However, our research question also implies an interest in how civil society actors are differently positioned within the political economy of social mobility. To offer insights here, we conducted a Latent Profile Analysis (LPA), a statistical technique which can be used to identify distinct groups of (in this case) organisations sharing similar behaviours or patterns of response across our nine dimensions (measured on the same three‐point scale) (Williams and Kibowski 2015). A key benefit of LPA is that this could help us distinguish coherent groupings even where individual coding scores appear ambiguous or hybrid. The LPA was estimated using Stata18. First, we estimated a series of latent profile models and calculated goodness of fit statistics (see Table 1). The 5‐profile model was preferred on account of the best overall fit with the data and the lowest values of AIC and BIC. Model estimates were produced for the five‐profile model, including the relative size of each latent profile expressed as a percentage (this is based on the prior probability). The profile for each latent group was then reported as a series of conditional mean scores based on the three‐point scale, where one indicates resistance to neoliberal logics, 2 reflects ambivalence or pragmatic alignment, and 3 signals reinforcement of neoliberal norms. Results are depicted in Figure 2, which maps and displays the average scores across nine dimensions for all five latent groups identified in the analysis. The chart captures marginal means for each group, providing an overview of how different clusters of organisations behave across the analytical dimensions. In Figure 2, darker tones correspond to stronger alignment with neoliberal governance, while lighter tones signal resistance.

**TABLE 1:** Latent profile models (goodness of fit statistics), *n* = 110.

Number of latent profiles	Log‐likelihood	d.f.	AIC	BIC
1	−643.78	18	1323.57	1372.17
2	−335.09	28	726.19	801.80
3	−227.50	38	531.01	633.63
4	−195.74	48	487.87	617.50
5	−155.74	58	427.49	584.12
6	−168.88	68	473.76	657.39

Abbreviations: AIC, Akaike Information Criteria; BIC, Bayesian Information Criteria.

## 2 Findings

### 2.1 Overview and Interpretation of Latent Profiles

Our initial data analysis revealed a marked pattern of ambivalence across the entire dataset. While some organisations exhibited clear tendencies either towards resistance or reproduction of neoliberal norms, the majority fell into a hybrid or intermediary position overall. This ambivalence could be interpreted as organisational indecision or conceptual vagueness, but our coding framework deliberately sought to foreground the structural conditions under which civil society operates, and the systemic functions of organisations. We suggest these results are therefore likely to reflect much wider structural ambivalence as, given the constraints imposed on civil society actors in neoliberal regimes, organisations comprising this social mobility industry are required to balance emancipatory values with institutional pragmatism. Ambivalence emerges then as a both descriptive category and an important insight, signalling the inevitable tensions and compromises that define how civil society is expected to manage inequality. The LPA further confirmed ambivalence as a systemic feature of this field, as it revealed five groups amongst the organisations we analysed. The distance between groups is narrow and arguably reflects variation in emphasis and strategy, rather than absolute ideological separation. Nevertheless, these differences are analytically significant as they show that, while the majority of organisations combine resistance with pragmatism, a small subset of organisations is more resistant towards co‐option and, at the other end of the scale, a distinct group more actively reproduces neoliberal governance, albeit with progressive branding. We approach these five groups as a typology and describe each in further detail below, with organisational examples.

### 2.2 Group 1: Structural Resistors

We call Group 1 ‘Structural Resistors’ as it includes charities and non‐profits that more actively contest neoliberal logic across nearly all dimensions. Organisations in this class scored consistently low (close to 1.0) on key variables including problem framing, civil society role, and governance diversity, indicating that they view inequality as systemic, position themselves as countervailing forces to market or state domination, and often involve beneficiary communities in leadership and decision‐making. Structural resistors tend to foreground collective emancipation over individual resilience and reflect civil society as a transformative space rather than a service delivery vehicle. Overall, they exhibit lower alignment with market logics, even if pragmatically dependent on external funding.

The 93% Club exemplifies this class. This is a charity established in 2016 by Sophie Pender, a first‐generation, state‐educated university student, explicitly motivated by her working‐class background. The charity's name references the c.93% of children educated by the state in the UK, who remain under‐represented in leading universities and elite professions. The charity's mission is to help tackle inequality and elitism by building a network for state‐schooled students and professionals, operationalised via peer‐led mentoring, career workshops and policy advocacy. This charity is not entirely outside neoliberal logics: for example, alongside membership and donations, it is funded through corporate partnerships. However, state education is framed within its own literature as a structural and cultural challenge, making this a systemic issue. The organisation is also student‐run from the ground‐up, with democratic elections and leadership from working‐class and first‐generation students. While the 93% Club operates within elite networks and market systems, it more actively challenges the legitimacy and exclusivity of private‐education pathways into top universities and careers, as illustrated by its tagline: ‘State School and Proud.’

These features make the 93% Club similar to other organisations within this class, which align most closely with the Polanyian ideal of civil society as a protective counter‐movement, confirming Fraser's argument that such resistance remains possible, if marginal. It is, though, important to note that Structural Resistors represented just 19% of our dataset and the strength of resistance is limited, since these organisations, like others across the social mobility industry, operate within a highly constrained policy and funding environment.

### 2.3 Group 2: Pragmatic Progressives

Group 2 was the largest group in our analysis, at almost 50% of our dataset. We name this group ‘Pragmatic Progressives’ as it contains organisations blending progressive intent and some structural awareness with institutional pragmatism. Organisations within this class typically score around 2.0 across most dimensions, indicating an ambivalent but broadly reformist position. They generally frame inequality as structural (problem framing mean = 1.0) and lean towards participatory or mixed models of expertise in terms of their leadership (mean = 1.28). However, they often rely on state or corporate funding and engage in forms of delivery that reflect performance‐oriented governance. While Structural Resistors affirm civil society is not uniformly co‐opted, Pragmatic Progressives work more obviously within dominant structures, demonstrating more institutional compliance as they translate their goals into fundable and scalable programmes. These two groups may therefore share some similar values but appear to differ around the degree of compromise considered palatable or perhaps necessary for survival by their leaders.

Charities such as the Social Mobility Foundation and The Brightside Trust are situated within this group, each of which supports young people from disadvantaged backgrounds into ‘elite’ universities and careers, in areas such as law, accountancy and medicine. This is achieved via interventions such as mentoring and coaching, skills training in areas such as interview technique and CVs, and the provision of internships and other forms of work experience. In their actions and words, these charities acknowledge systemic barriers to opportunity, such as unequal access to elite professions, cultural capital, and educational networks, and frame inequality in structural or socio‐economic terms. However, they pursue change *within* existing institutional frameworks. For example, The Social Mobility Foundation (SMF) seeks to remedy class‐based exclusion from elite professions by connecting talented students to elite employers, suggesting reform predominantly through access, rather than structural overhaul of elite labour markets. The Brightside Trust uses mentoring to build confidence and decision‐making skills in young people, while emphasising evaluation, impact metrics, and partnership with higher education institutions.

This is undoubtedly a pragmatic approach. Progressive structural intent is balanced against institutional dependency, including on powerful and elite organisations funding their work and supporting their students and alumni. Critically, however, organisations in this class do not challenge the legitimacy of those elite institutions, or wider employment hierarchies. Pragmatic Progressives therefore illustrate how counter‐movements are not entirely extinguished but are absorbed into the system they seek to correct. Once again, this aligns with Fraser's extension of Polanyi, where the protective functions of civil society can be co‐opted not to *resist* commodification but to help stabilise it, ultimately functioning towards system stability rather than system change.

### 2.4 Group 3: Technocratic Deliverers

We call Group 3 ‘Technocratic Deliverers’, as organisations most closely integrated with the state, often functioning as contracted agents. While they often frame inequality as structural (problem framing = 1.0), their mode of intervention is typically heavily managerial, built around metrics, targets, and outcome reporting.

Members of this group include Teach First and the Social Mobility Pledge, both of which represent this distinctive organisational form, which is not radically neoliberal in language or positioning but is structurally aligned with the governing logics of marketized public service. These charities have different aims, but both deliver policy in close partnership with government or corporate actors. For example, Teach First, a charity which recruits and trains graduates to teach in disadvantaged schools, aims to address educational inequality through leadership development. Teach First has deep ties to government departments and large‐scale public contracts, including with the Department for Education. Its primary intervention is focussed on teacher placement in disadvantaged schools, an outcomes‐based delivery model, framed around leadership, impact, and school performance. It uses evaluation frameworks and KPIs common to marketised governance, even as it acknowledges educational inequality. The Social Mobility Pledge works at the policy–employer interface, encouraging institutions to commit to voluntary reforms around outreach, recruitment, and workplace inclusion. It frames social mobility as a moral and economic imperative for business, using the language of ‘fairness,’ ‘talent’ and ‘opportunity’, without explicitly critiquing structural power or inequality. These organisations do not frame themselves as part of civil society resistance, but rather as delivery partners or facilitators of institutional change, shaped by technocratic and performative rationalities.

This was the smallest of our five groups, comprising just 6 organisations, which might be considered outliers compared to others in our dataset, given their specific relation to the state. They are though important to include in our analysis, and understand, as they often fill gaps left by public sector withdrawal, particularly in education, tutoring, or employability. As they substitute for state capacity, these organisations tend to reframe social problems as *technical* issues to be solved through management, leadership, and employer best practice. This suggests that ‘Technocratic Deliverers’ exemplify Fraser's critique of the co‐option of civil society, as organisations which may acknowledge inequality, but which operate as governance tools nevertheless, rather than distinct counter‐movements.

### 2.5 Group 4: Professionalised Reformers

Group 4 includes organisations operating with something of a reformist ethos, yet which are more fully embedded in the professionalised logic of social service delivery. As such, we name this class: ‘Professionalised Reformers.’ Their scores cluster around 2.0 on most dimensions, indicating some ambivalence but edging towards stronger alignment with neoliberal norms in dimensions such as their use of language (2.18) and problem framing (2.06). Both relate to a tendency to describe structural inequality through relatively apolitical and individualistic terms.

The Sutton Trust and Causeway Education are examples of Professionalised Reformers. The former works to improve social mobility via evidence‐based programmes and advocacy, focussing on access to elite universities and professions for young people from disadvantaged backgrounds, while the latter supports students from underrepresented groups in making informed post‐16 and university choices, primarily through targeted guidance, application support, and collaboration with teachers and schools. Both recognise social inequality, particularly in access to education or elite employment. They often cite data on underrepresentation, school disadvantage, or socioeconomic background as motivations for their interventions. However, these charities tend to frame these issues through terms that are measurable and might be considered particularly ‘funder‐legible,’ such as ‘skills gaps,’ ‘confidence,’ ‘aspiration,’ and ‘work readiness.’ A related feature is as they design structured interventions that are outcome‐driven, often with reportable impact data. e.g., the Sutton Trust is known for its evidence‐based reports and advocacy but also runs highly selective social mobility pathways (e.g. Pathways to Law), framed around gaining access to elite universities and professions. Causeway Education builds tools and teacher support systems to improve post‐16 progression but focuses most obviously on technical fixes, such as application writing and decision‐making, rather than challenging systemic barriers.

Professional Reformers differ from Technocratic Deliverers as they typically operate with greater distance from the state. While the latter often substitute for public services and are embedded in state funding, Professional Reformers tend to work adjacent to government, using evidence and advocacy to influence systems while still conforming to their rules. Overall, they exhibit a type of ‘progressive *performance*,’ which may help wider systems look like they are working towards fair outcomes, without disrupting structural inequalities. While Professionalised Reformers share some goals with Pragmatic Progressives, they occupy a distinctive space within the field. For example, the latter balance critical framing with tactical compliance, while Professionalised Reformers are more fully aligned with professional delivery norms. Their strategies are also less participatory and more expert‐led, often relying on grant models, programme evaluation, and formalised toolkits to deliver scalable interventions. Professionalised Reformers also differ from Structural Resistors, as the latter centre mobilisation from the ground‐up, along with lived experience, and political critique, while the former work through established institutions and often seek legitimacy through policy influence, employer partnerships, and data‐driven accountability. While not a large group, at 15% of the total, Professionalised Reformers illustrate how civil society can perform equity while leaving underlying hierarchies intact, showing once again how the mechanisms of social mobility can stabilise inequality under the guise of reform.

### 2.6 Group 5: System‐Conforming

Group 5 includes the organisations most aligned with neoliberal governance, scoring above 2.5 on dimensions such as market conformity (3.0), neoliberal language (2.95), funding model (2.74), and role of expertise (2.84). As such, we call this group ‘System‐Conforming.’ These organisations are most likely to frame inequality in market‐compatible terms, as a failure of opportunity, talent pipelines, or individual readiness, with programmes heavily shaped by the logics of performance, employability, and corporate partnership. They often maintain strong links to elite institutions or business networks, using these connections as both a resource and arguably, a marker of legitimacy.

The Brilliant Club and SEO London exemplify this group. SEO London prepares ‘talented’ students from underserved backgrounds for careers in elite industries, such as finance, law and consulting, through training, mentoring, and access to professional networks designed to help them succeed within highly competitive sectors. It offers training to ensure they ‘fit’ within professional cultures, and celebrates those who get in, reinforcing narratives of personal resilience and exceptionalism. The Brilliant Club aims to increase the number of pupils from underrepresented backgrounds progressing to highly selective universities by placing researchers in schools to deliver academic enrichment programmes, fostering university‐style learning and raising aspirations among students who may not otherwise consider or access elite higher education. As it prepares students to access existing structures of privilege there is no attempt to reform those structures, with one result that higher education's role as a legitimate sorting mechanism for social mobility is reinforced. Overall, these charities play a *functionary* role, helping to smooth the path for students from less advantaged backgrounds into elite institutions while failing to contest what offers those institutions power to act as gatekeepers, and tending to reward individual conformity to neoliberal professionalism.

System‐Conformers were the third largest group, at 19 organisations and 17% of the total. They are most clearly differentiated from Structural Resistors as they are deeply embedded in the system they might otherwise critique. While Structural Resistors frame inequality as structural (mean ≈ 1.0), System‐Conformers are likely to frame it in more behavioural or depoliticised terms (mean > 2.0). The role of expertise also especially divides these groups, with Structural Resistors favouring participatory or community‐led knowledge while System‐Conformers rely on technocratic, KPI‐oriented logics. At first glance, System‐Conformers have more in common with Pragmatic Progressives. They are, though, less participatory and more delivery‐focused, with a more highly professionalised and explicitly performance driven profile. Overall, this group arguably offers the clearest case of civil society operating not as a counter‐movement but as a legitimising infrastructure for neoliberal order, as they rhetorically co‐opted, and structurally aligned with state functions. They show how civil society can become an extension of technocratic governance, not resisting neoliberalism but managing its contradictions, offering especially clear evidence of how civil society can serve to stabilise the very inequalities it claims to confront.

## 3 Discussion

This article set out to examine how civil society actors, particularly charities and non‐profit organisations operating in the UK social mobility space, negotiate their role within neoliberal governance. Drawing on Polanyi's (1944) concept of the double movement and Fraser's (2011, 2014, 2017) critique of marketised social protections, we asked whether these organisations operate as counter‐movements resisting commodification and/or as institutional mechanisms that help to stabilise the very inequalities they are meant to address.

Our findings reveal a field structured by deep ambivalence, which we suggest is in fact a dominant mode of social action under neoliberal governance. The majority of organisations we analysed fell into intermediate positions across our Fraserian coding dimensions, neither clearly resisting nor fully reproducing neoliberal logics. Rather than indicating conceptual vagueness or analytical indecision, we argue this pattern reflects the structural constraints under which civil society operates, and confirms that ambivalence is an underlying condition and perhaps requirement of civil society under neoliberalism, rather than an exception. Latent Profile Analysis clarified how this ambivalence plays out in practice. This identified five distinct organisational groups, ranging from Structural Resistors, a small cohort characterised by more ‘ground‐up’ governance, community‐led expertise, and a more marked oppositional stance, through to System‐Conformers at the other end of the scale, whose framing, funding, and delivery modes align more fully with market rationalities. Most organisations fell into the middle: Pragmatic Progressives work within dominant structures while trying to bend them towards equity while Professionalised Reformers and Technocratic Deliverers stabilise the system through policy‐aligned, performance‐driven interventions.

We underline once more that the differences between these groups are relatively small and boundaries between them may be blurred. However, we also found that the strongest differentiators between groups were how organisations frame inequality, as either structural or individual and behavioural, how they use expertise, in ways which are either community led or more technocratic, and how they define their civil society role, as oppositional or delivery oriented, or somewhere in between. These findings are significant as they reveal how, in the face of shared constraints, these organisations understand injustice somewhat differently, and vary on questions such as whose knowledge they centre, and whose interests they ultimately serve. Nevertheless, it is also notable that Group 2, ‘Pragmatic Progressives,’ was by far the largest group, comprising nearly half the dataset, suggesting that the dominant survival strategy in contemporary civil society is to balance critique and compliance. Organisations in this class speak the language of justice but we suggest are structurally required to frame their work through scalable, funder‐friendly terms. This means they negotiate their values within a field shaped by institutional logics or what Fraser (2011) might call a ‘contained critique.’ For so many organisations to fall into this category underscores how resistance is not eliminated under neoliberal governance but is, on the other hand, closely controlled.

Our study makes three original contributions. Empirically, we provide the first systematic mapping and typology of the UK's social mobility industry (Payne 2017, Todd 2021), capturing both its ideological variation and structural coherence. Theoretically, this allows us to extend Polanyi's (1944) and Fraser (2011, 2017) insights into a specific and influential policy field, showing how the latter's ‘marketised social protections’ not only fail to deliver justice, but also help to re‐legitimise market logic through progressive optics. We add nuance to Fraser's (2011) critique, to highlight some potential for resistance. Rather than suggesting a binary position where civil society either mitigates or reproduces neoliberal inequality, our analysis reveals a more complex picture. Nevertheless, while most organisations adopt structural framings of inequality, these are translated into individualised, funder‐aligned interventions shaped by performance logics, technocratic language, and institutional dependency. Overall, civil society organisations in the UK social mobility industry largely operate not as counter‐movements resisting marketisation, but as institutional actors negotiating its terms.

Our third contribution is methodological, as the use of Latent Profile Analysis alongside qualitative coding, to underline once more that within the social mobility industry ambivalence is structurally produced, as organisations tasked with redressing inequality tend to perform inclusion without redistributing power. Here, we identify a typology that confirms civil society's role is not fixed but fractured, ranging from structural resistance to neoliberal legitimation, and suggest that this offers a scalable way to diagnose organisational orientation within constrained systems.

We believe our study can usefully inform future research in several important ways. First, our methodological approach combining Latent Profile Analysis and qualitative coding offers a tool researchers might use to map power, governance, and resistance within other policy arenas. Second, our analysis has focussed primarily on how civil society organisations negotiate neoliberal governance in relation to class, market logic, and structural inequality, with limited attention to race as an intersecting axis of injustice. However, while race was not explicitly foregrounded in our coding framework, we note that organisations focussed on racial justice are disproportionately represented in the most resistant cluster, suggesting anti‐racist practice may provide a particularly powerful basis for resisting co‐option and reframing social mobility in more transformative terms. We believe this is an important subject for future research, as organisations especially focussed on racial justice may adopt similar structural critiques to those grounded in class‐based analysis, but they often do so with a distinct historical consciousness, community orientation, and epistemic challenge to dominant institutions.

Third, our analysis was based on publicly available websites and linked documents (including strategy pages, annual reports, and impact statements). While offering strong insights into organisations' current activities, practices and aims, this picture is also relatively static and we could not trace how such activities have evolved over time, possibly in step with the spread of neoliberalism. A related insight is though, that organisations established more recently were more likely to be found in Group 5, ‘system‐conformers’, which could suggest that those with a longer history are more able to resist. The type of historical analysis required to test these assertions was beyond the scope of the current study but again, could provide more in‐depth insights, including how organisational missions, language, funding models, and programmatic strategies have shifted in response to policy changes, funding regimes, and dominant ideologies. Such an approach could help distinguish between foundational values and adaptive practices, revealing whether apparent resistance is a legacy of earlier institutional arrangements or a sustained organisational commitment. It would also allow researchers to trace the temporal embedding of neoliberal logics examining, for example, whether earlier commitments to redistribution or participatory governance have been reframed through managerial, skills‐based, or outcomes‐led vocabularies. In this sense, a historical lens could reveal the longitudinal dynamics of co‐option, adaptation, or resilience that are only partially visible in cross‐sectional, present‐day data, as presented here.

Fourth, though closely related, it is also important to recognise that these materials reflect a curated, outward‐facing image of each organisation. Such representations are often shaped by funding imperatives, reputational concerns, and institutional norms, and may not fully reflect the underlying beliefs, values, or practices of those working within these organisations. In this sense, our analysis captures how organisations choose, or perhaps feel compelled, to present themselves within a competitive and audit‐driven environment. This offers important insights into the structural pressures and discursive expectations that shape civil society under neoliberal governance, but it also introduces limitations. Specifically, it could over‐represent technocratic or market‐oriented framings, even where individuals within organisations may hold more critical or emancipatory views. We therefore view this public‐facing data as revealing of institutional positioning rather than personal intent, and advocate for future qualitative research, particularly interviews with staff and leaders, to explore how actors within these organisations navigate tensions between structural critique and institutional survival.

This is an important goal since, while not intentional, the current social mobility industry makes symbolic reform appear as meaningful action, particularly to policy actors, donors, and even some critical observers, while preserving the legitimacy of a profoundly unequal system (Ashley 2022). We argue this is one way in which neoliberalism patches over its own contradictions, preventing public dissent or even anger, which could contribute to more radical demands for structural change. While previous analyses have described the failures of social mobility policy (Ingram and Gamsu 2022, Mountford‐Zimdars et al. 2024), we have focussed more closely on *how* failure is made part of its function, as the associated industry manages inequality in ways that divert attention from structural redistribution, offering hope without transformation. Explaining the mechanisms behind this process is vital, because it illuminates how civil society actors become entangled in the reproduction of inequality, helping both practitioners and academics understand the limits of current approaches and recognise the pressures shaping their work. Potentially, this could also help those concerned with inequality consider where spaces might exist for more radical or emancipatory practice, and how these could be leveraged.

## Ethics Statement

Our analysis relied solely on publicly available information drawn from organisational websites and did not involve human participants or private data. While not subject to formal institutional review, we took ethical considerations seriously throughout. In particular, we recognise potential risks associated with our analysis, specifically if our critique of wider systems could suggest organisational reputational harm. To mitigate this risk, we have anonymised findings at the aggregate level in our typology (which is available on request). While we use exemplar organisations as illustrative, we underline here and in the paper that our critique in no way implies failure or blame. Instead, organisational actions are interpreted as responses to wider structural constraints and funding pressures, and not individual or institutional shortcomings.

## Conflicts of Interest

The authors declare no conflicts of interest.

## Data Availability

The data that support the findings of this study are available on request from the corresponding author. The data are not publicly available due to privacy or ethical restrictions.

## Endnotes

^1^
The full list including how organisations were categorised in our analysis is available on request from the corresponding author.
